# Involvement of Ferroptosis Induction and Oxidative Phosphorylation Inhibition in the Anticancer-Drug-Induced Myocardial Injury: Ameliorative Role of Pterostilbene

**DOI:** 10.3390/ijms25053015

**Published:** 2024-03-05

**Authors:** Kiyomu Fujii, Rina Fujiwara-Tani, Shota Nukaga, Hitoshi Ohmori, Yi Luo, Ryoichi Nishida, Takamitsu Sasaki, Yoshihiro Miyagawa, Chie Nakashima, Isao Kawahara, Ruiko Ogata, Ayaka Ikemoto, Rika Sasaki, Hiroki Kuniyasu

**Affiliations:** Department of Molecular Pathology, Nara Medical University, 840 Shijo-cho, Kashihara 634-8521, Nara, Japan; toto1999-dreamtheater2006-sms@nifty.com (K.F.); shota.nukaga@gmail.com (S.N.); brahmus73@hotmail.com (H.O.); lynantong@hotmail.com (Y.L.); g.m_r1@outlook.jp (R.N.); takamitu@fc4.so-net.ne.jp (T.S.); y.miya1103@gmail.com (Y.M.); c-nakashima@naramed-u.ac.jp (C.N.); isao_kawahara@a011.broada.jp (I.K.); pkuma.og824@gmail.com (R.O.); a.ikemoto.0916@gmail.com (A.I.); rika0st1113v726296v@icloud.com (R.S.)

**Keywords:** drug-induced cardiac damage, ferroptosis, mitochondrial damage, mitochondrial iron, pterostilbene, antioxidants, chemical prevention

## Abstract

Patients with cancer die from cardiac dysfunction second only to the disease itself. Cardiotoxicity caused by anticancer drugs has been emphasized as a possible cause; however, the details remain unclear. To investigate this mechanism, we treated rat cardiomyoblast H9c2 cells with sunitinib, lapatinib, 5-fluorouracil, and cisplatin to examine their effects. All anticancer drugs increased ROS, lipid peroxide, and iron (II) levels in the mitochondria and decreased glutathione peroxidase-4 levels and the GSH/GSSG ratio. Against this background, mitochondrial iron (II) accumulates through the unregulated expression of haem oxygenase-1 and ferrochelatase. Anticancer-drug-induced cell death was suppressed by N-acetylcysteine, deferoxamine, and ferrostatin, indicating ferroptosis. Anticancer drug treatment impairs mitochondrial DNA and inhibits oxidative phosphorylation in H9c2 cells. Similar results were observed in the hearts of cancer-free rats treated with anticancer drugs in vitro. In contrast, treatment with pterostilbene inhibited the induction of ferroptosis and rescued the energy restriction induced by anticancer drugs both in vitro and in vivo. These findings suggest that induction of ferroptosis and inhibition of oxidative phosphorylation are mechanisms by which anticancer drugs cause myocardial damage. As pterostilbene ameliorates these mechanisms, it is expected to have significant clinical applications.

## 1. Introduction

Cancer cachexia occurs in approximately 80% of patients with advanced cancer [1]. Myocardial damage associated with cancer cachexia is the second leading cause of death among patients with cancer [2]. Myocardial dysfunction in patients with cancer makes treatment continuation difficult and worsens life expectancy. Therefore, elucidating the mechanisms underlying cancer-induced myocardial dysfunction and developing methods to ameliorate this dysfunction are important for cancer treatment.

Much of the focus has been on cancer-therapy-related cardiac dysfunction (CTRCD) caused by anticancer drugs [3,4]. The best-understood mechanism of myocardial injury from anticancer drugs is caused by doxorubicin (DOX); more than 25% of patients receiving a cumulative dose of 550 mg/m^2^ DOX develop congestive heart failure [5]. Cardiotoxicity is another concern with anticancer drugs other than anthracyclines [6]. The accumulation of lipid peroxide and reduction in the expression of glutathione (GSH) and glutathione peroxidase-4 (GPX4) observed in DOX-induced mitochondrial iron (II) (mtFe) accumulation suggest that ferroptosis occurs in the myocardium [7]. However, DOX is not currently a first-line drug in many cancers. Here, we investigated the cardiotoxicity of 5-fluorouracil (5FU), cisplatin (CDDP), sunitinib (SUN), and lapatinib (LAP), which are used as main therapeutic agents for many cancers [8]. Cardiotoxicity has been reported with these drugs, but the details are still unclear [9,10,11,12].

Ferroptosis is a unique mode of cell death that is mechanistically and morphologically distinct from other cell deaths, such as apoptosis, as it is iron-dependent and is caused by the toxicity of accumulated lipid peroxide in the cell membrane [13,14]. Ferroptosis is an important process that mediates the etiology and progression of many cardiovascular diseases, including atherosclerosis, drug-induced heart failure, myocardial ischemia–reperfusion injury, sepsis-induced cardiomyopathy, arrhythmias, and diabetic cardiomyopathy [15]; ferroptosis is also involved in DOX-induced cardiac injury [16]. In this study, we examined the effects of various anticancer drugs on ferroptosis induction in cardiomyocytes.

Pterostilbene (PTE) is a well-known antioxidant found primarily in blueberries [17]. PTE has various pharmacological properties, including chemopreventive, anti-inflammatory, anti-diabetic, anti-lipidemic, anti-atherosclerotic, and neuroprotective properties [18]. Myocardium inhibits myocardial fibrosis [19], reduces ischemia–reperfusion-induced inflammation [20], and prevents hypertensive heart failure [21,22]. The inhibition of oxidative stress [23,24] reduced lipid peroxidation, maintained thioredoxin reductase activity, promoted GST and glutaredoxin activity, and reduced nuclear factor erythroid 2 (Nrf2) and AMP-activated protein kinase (AMPK) activation [25,26,27]. However, reports on the effects of PTE on CTRCD are limited.

In this study, after elucidating the effects of various anticancer drugs on the myocardium, we investigated the myocardial protective effects of PTE in CTRCD.

## 2. Results

### 2.1. Effects of Anticancer Drugs on H9c2 Cells

**Figure 1:**
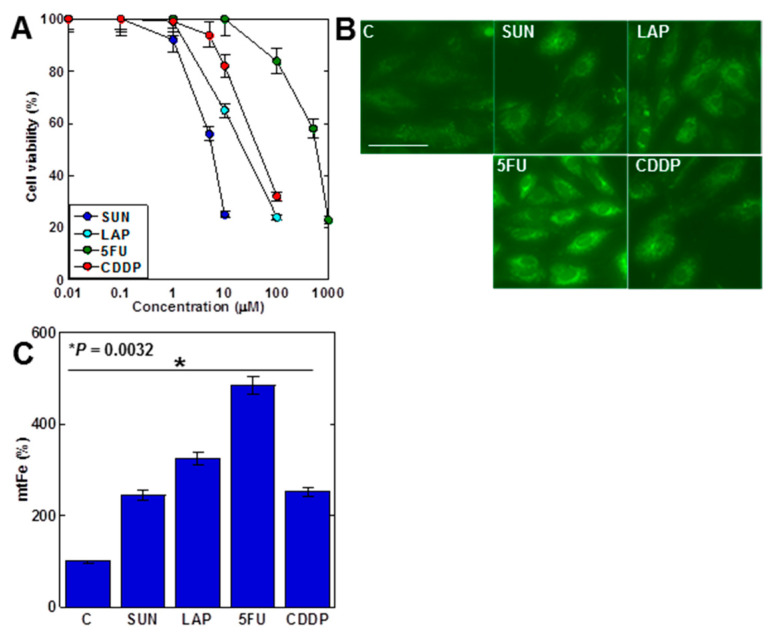
Effects of anticancer drugs in H9c2 myocardial cells. (**A**) Effects of four anticancer drugs on cell viability in H9c2 cells. (**B**) Effects of anticancer drugs (IC_20_) on mtFe accumulation. (**C**) Semi-quantification of mtFe. Error bars represent the standard deviations from three independent trials. Statistical difference was calculated by AVOVA. Scale bar, 20 μm. SUN, sunitinib; LAP, lapatinib; 5FU, 5-fluorouracil; CDDP, cisplatin; mtFe, mitochondrial iron (II).

First, we examined the effects of four anticancer drugs on H9c2 cell proliferation (Figure 1A). All anticancer drugs inhibited cell growth in a dose-dependent manner. Both SUN and LAP, which showed mtFe deposition in gastric cancer, and 5FU and CDDP, which did not show mtFe deposition [28,29], showed mtFe deposition in H9c2 cells (Figure 1B,C).

### 2.2. Redox Alteration in H9c2 Cells Induced by Anticancer Drugs

**Figure 2:**
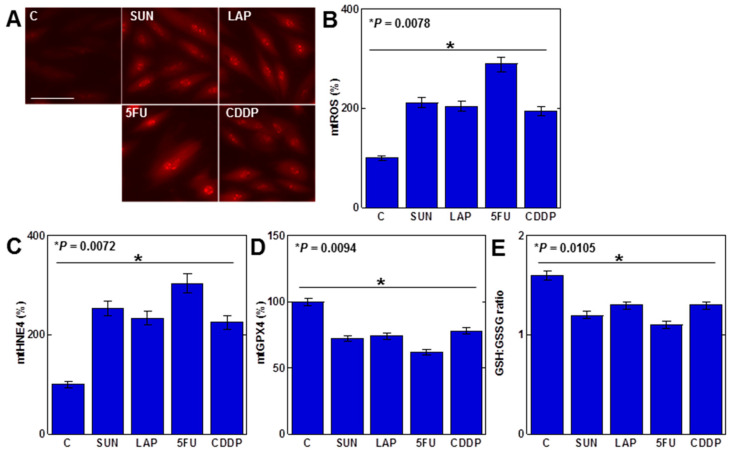
Effects of anticancer drugs on mitochondrial redox in H9c2 cells. (**A**) Effects of anticancer drugs (IC_20_) on mtROS production. (**B**) Semi-quantification of mtROS. (**C**,**D**) Mitochondrial levels of 4HNE (**C**) and GPX4 (**D**). (**E**) GSH/GSSG ratio. Error bar, standard deviation from three independent trials. Statistical difference was calculated by AVOVA. C, control; SUN, sunitinib; LAP, lapatinib; 5FU, 5-fluorouracil; CDDP, cisplatin; mtROS, mitochondrial hydroxy radical; mt4HNE, mitochondrial 4-hydroxynonenal; mtGPX4, mitochondrial glutathione peroxidase-4; GSH, glutathione; GSSG, glutathione disulfide.

As mtFe deposition was observed with all anticancer drugs, we next examined the redox alterations induced by these drugs (IC_20_) (Figure 2). Levels of mitochondrial hydroxyl radicals (mtROS) were increased by all anticancer drugs (Figure 2A,B). When mitochondria were extracted and examined, levels of mitochondrial lipid peroxide (4-hydroxynonenal, 4HNE) were also increased by all anticancer drugs (Figure 2C). In contrast, mitochondrial GPX4 expression and the GSH/GSSG ratio decreased (Figure 2D,E).

### 2.3. Induction of Cell Death by Anticancer Drugs in H9c2 Cells

**Figure 3:**
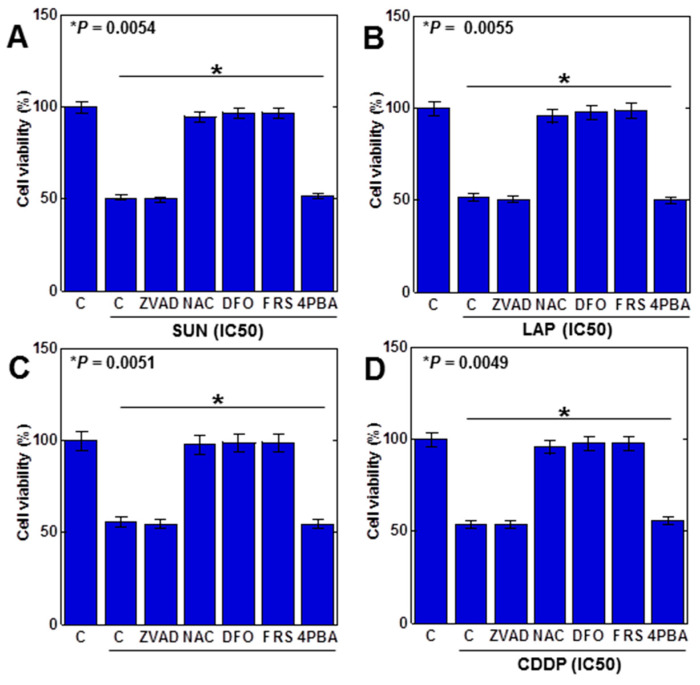
Induction of cell death by anticancer drugs in H9c2 cells. H9c2 cells were treated with four anticancer drugs (IC_50_) for 24 h in combination with various cell death inhibitors. (**A**) SUN, (**B**) LAP, (**C**) 5FU, (**D**) CDDP. Error bar, standard deviation from three independent trials. Statistical difference was calculated by AVOVA. SUN, sunitinib; LAP, lapatinib; 5FU, 5-fluorouracil; CDDP, cisplatin; C, control; ZVAD, Z-VAD-FMK; NAC, N-acetyl-L-cysteine; DFO, deferoxamine; FER, ferrostatine-1; 4PBA, 4-phenylbutyric acid.

The above results suggested that ferroptosis was induced by the anticancer drug. Therefore, we examined the viability of anticancer (IC_50_) drugs in H9c2 cells with various cell death inhibitors (Figure 3). Decreased viability by SUN was rescued by N-acetylcysteine (NAC, antioxidant), deferoxamine (DFO, iron chelator), and ferrostatin 1 (FRS, a ferroptosis inhibitor) but not by Z-VAD-FMK (ZVAD; apoptosis inhibitor) or 4-phenylbutyric acid (4PBA, an endoplasmic reticulum stress inhibitor), suggesting that SUN induces ferroptosis in H9c2 cells (Figure 3A). Similar results were observed for LAP, 5FU, and CDDP (Figure 3B–D). These results suggested that all anticancer drugs induced ferroptosis in H9c2 cells.

### 2.4. MtFe Deposition by Anticancer Drugs in H9c2 Cells

**Figure 4:**
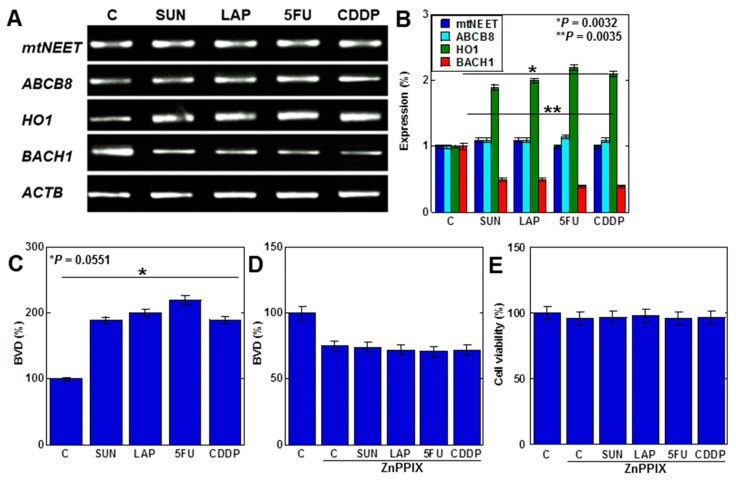
Effects of anticancer drugs on mtFe accumulation. (**A**) Effects of anticancer drugs (IC_20_) on mRNA expression of mtFe-associated genes. (**B**) Semi-quantification of RT-PCT signals. (**C**) Effect of anticancer drugs on BVD production. (**D**,**E**) Effect of ZnPPIX on BVD production (**D**) and cell viability (**E**). Error bar, standard deviation from three independent trials. Statistical difference was calculated by AVOVA. SUN, sunitinib; LAP, lapatinib; 5FU, 5-fluorouracil; CDDP, cisplatin; C, control; mtFe, mitochondrial iron (II); RT-PCR, reverse transcription–polymerase chain reaction; mtNEET, mitochondrial protein containing the Asn-Glu-Glu-Thr (NEET) sequence; ABCB8, ATP-binding cassette subfamily B member 8; HO1, haem oxygenase-1; FCH, ferrochelatase; ZnPPIX, zinc protoporphyrin IX; BVD, biliverdin.

We next investigated the mechanism of mtFe deposition (Figure 4). We investigated mitochondrial iron transporter molecules, mitochondrial proteins containing the Asn-Glu-Glu-Thr (NEET) sequence (mtNEET), and ATP-binding cassette subfamily B member 8 (ABCB8) as causes of mtFe deposition. The anticancer drugs showed no changes in gene expression of mtNEET and ABCB8 (Figure 4A,B). In contrast, haem oxygenase-1 (HO1), which degrades haem and increases mtFe levels, was upregulated (Figure 4A,B). The expression of ferrochelatase (FCH), which promotes haem synthesis, was also upregulated. The level of haem degradation, as measured by the amount of biliverdin (BVD) that is a byproduct of the HO1 enzymatic reaction, increased with anticancer drugs (Figure 4C). Furthermore, inhibition of HO1 activity by zinc protoporphyrin IX (ZnPPIX) inhibited BVD production and cell death in H9c2 cells with anticancer drugs (Figure 4D,E).

These findings suggest that anticancer drugs promote haem degradation by enhancing mitochondrial HO1 expression in cardiomyocytes, resulting in mtFe accumulation.

### 2.5. Anticancer Drugs Inhibit Mitochondrial DNA (mtDNA) Damage and Oxidative Phosphorylation (OXPHOS)

**Figure 5:**
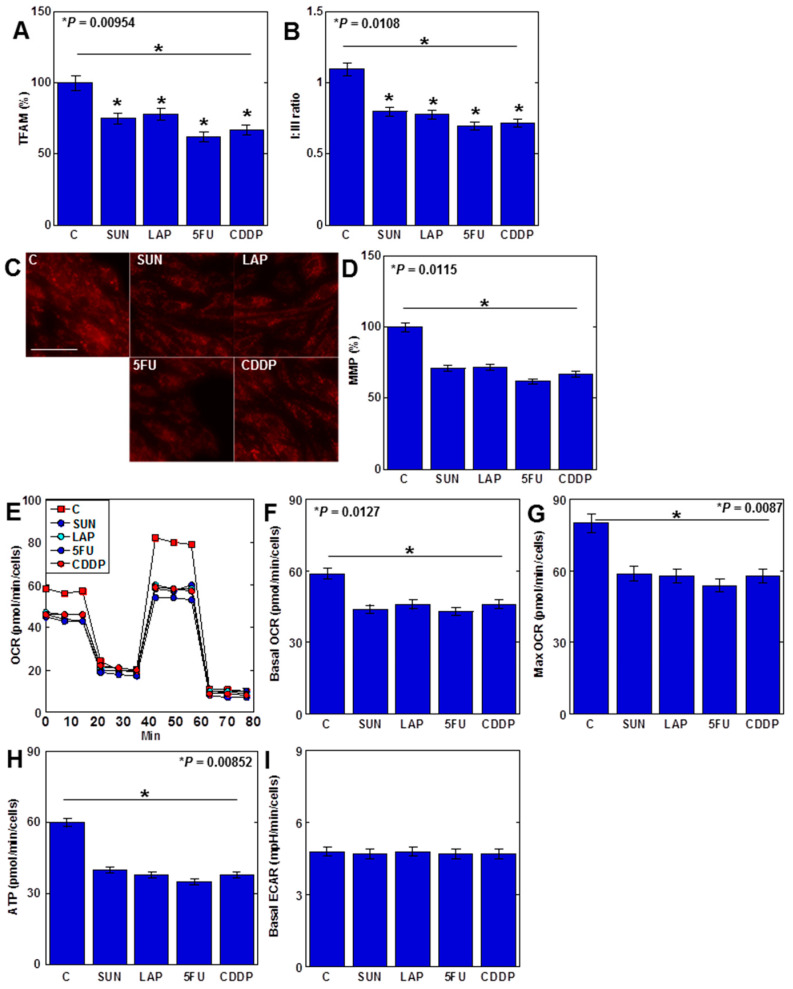
Effects of anticancer drugs on mtDNA damage and suppression of OXPHOS. (**A**–**D**) Effects of four anticancer drugs (IC20) on TFAM protein levels (**A**), C-I/C-III ratio (**B**), and MMP (**C**) and its semi-quantification (**D**). (**E**–**I**) Flux analysis of anticancer-drug-treated H9c2 cells: (**E**) time course, (**F**) basal OCR, (**G**) maximum OCR, (**H**) ATP, and (**I**) ECAR. Error bar, standard deviation from three independent trials. Statistical difference was calculated by AVOVA. SUN, sunitinib; LAP, lapatinib; 5FU, 5-fluorouracil; CDDP, cisplatin; C, control; OXPHOS, oxidative phosphorylation; TFAM, mitochondrial transcription factor A; C-I, electron transfer chain complex I; C-III, electron transfer chain complex III; MMP, mitochondrial membrane potential; OCR, oxygen consumption rate; ECAR, extracellular acidification rate.

Anticancer drugs increased mtROS levels, and their specific effects on the mitochondria were then examined (Figure 5). MtDNA protein mitochondrial transcription factor A (TFAM) levels in H9c2 cells decreased after treatment with all four anticancer drugs (Figure 5A). When the protein ratio of electron transfer chain complexes I and III (C-I/C-III ratio) was examined, it decreased with all four drugs (Figure 5B). Furthermore, the mitochondrial membrane potential (MMP) of H9c2 cells was reduced by all anticancer drugs (Figure 5C,D). In the flux assay, the basal oxygen consumption ratio (OCR) and maximum OCR, as well as APT production, were reduced with all four drugs (Figure 5E–H). In contrast, the extracellular acidification rate (ECAR) levels remained unchanged (Figure 5I).

Taken together, these findings suggest that anticancer-drug-induced mtDNA damage results in the inhibition of OXPHOS and impairment of myocardial energy metabolism. This also suggests that anticancer drugs have a dual inhibitory effect on cardiomyocytes by inducing ferroptosis and suppressing energy metabolism.

### 2.6. Effects of PTE on Cell Protection in H9c2 Cells

**Figure 6:**
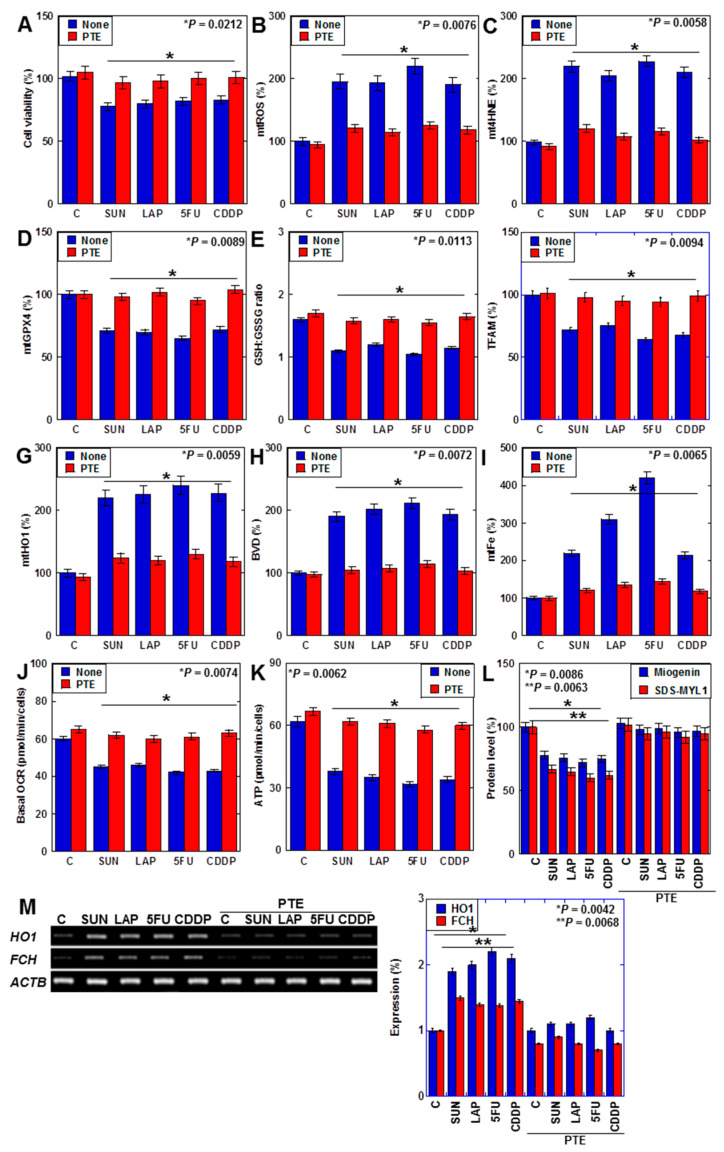
Effects of PTE on cell protection in H9c2 cells. (**A**–**L**) Effects of PTE in anticancer drug (IC_20_)-treated H9c2 cells on cell viability (**A**), mtROS (**B**), mt4HNE (**C**), mtGPX4 (**D**), GSH/GSSG ratio (**E**), TFAM (**F**), mtHO1 (**G**), BVD production (**H**), mtFe (**I**), basal OCR (**J**), ATP (**K**), myogenin and SDS-MYL1 (**L**), mRNA expression of HO1 and FCH (**M**), and its semi-quantification. Error bar, standard deviation from three independent trials. Statistical difference was calculated by AVOVA. PTE, pterostilbene; SUN, sunitinib; LAP, lapatinib; 5FU, 5-fluorouracil; CDDP, cisplatin; C, control; mtROS, mitochondrial hydroxy radical; mt4HNE, mitochondrial 4-hydroxynonenal; mtGPX4, mitochondrial glutathione peroxidase-4; GSH, glutathione; GSSG, glutathione disulfide; TFAM, mitochondrial transcription factor A; HO1, haem oxygenase-1; FCH, ferrochelatase; BVD, biliverdin; mtFe, mitochondrial iron (II); OCR, oxygen consumption rate; SDS-MYL1, sodium dodecyl sulfate-soluble myosin light chain-1.

We previously reported that PTE improves SUN-induced myocardial atrophy [29]. In this study, we investigated the effects of PTE on myocardial damage induced by SUN, LAP, 5FU, and CDDP (Figure 6). PTE restored the viability of cells that were treated with all four drugs (Figure 6A). Additionally, although all anticancer drugs increased mtROS and mt4HNE levels, decreased GPX4 expression, decreased the GSH/GSSG ratio, and decreased TFAM levels in H9c2 cells, these were restored by PTE (Figure 6B–F). Moreover, anticancer-drug-induced increases in mitochondrial HO1 expression, biliverdin levels, and mtFe deposition were attenuated by PTE (Figure 6G–I). In the flux assay, OCR and ATP production were reduced by anticancer drugs but were improved by PTE (Figure 6J,K). Levels of myogenin and sodium dodecyl sulfate-soluble myosin light chain-1 (SDS-MYL1), markers of myocardial maturation, were also decreased by anticancer drugs but recovered by PTE (Figure 6L). Furthermore, anticancer-drug-induced upregulation of FCH expression was attenuated by PTE treatment (Figure 6M).

Taken together, PTE attenuated anticancer-drug-induced mitochondrial damage by suppressing ROS and downregulating HO1 expression in cardiomyocytes.

### 2.7. Effects of PTE on Anticancer-Drug-Induced Myocardial Damage in Rats

**Figure 7:**
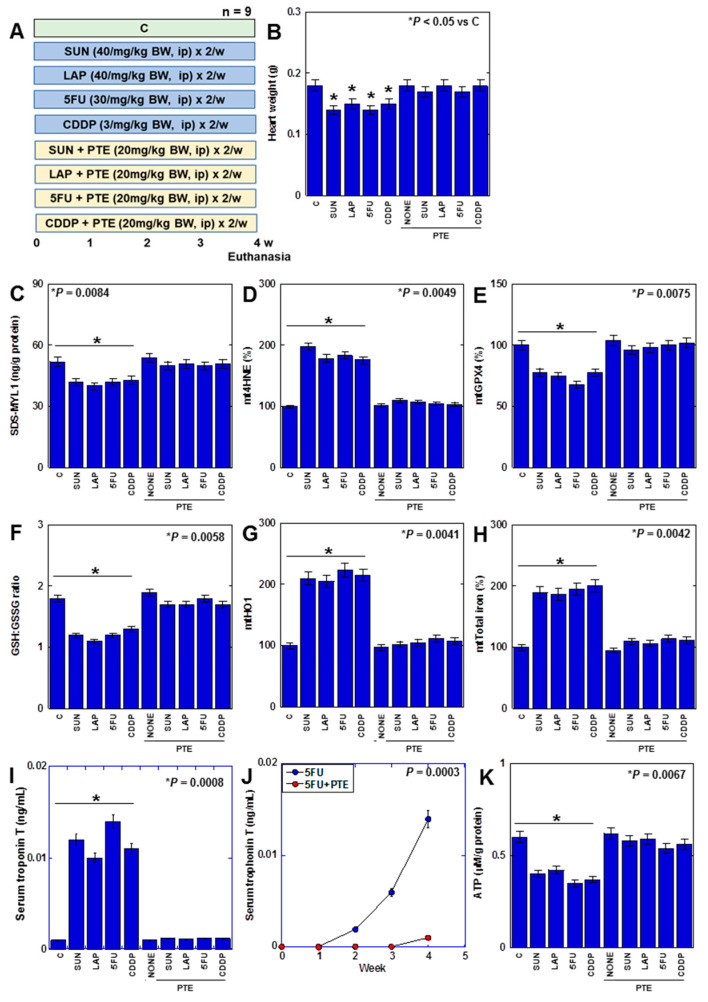
Effects of PTE on anticancer-drug-induced myocardial damages in rats. (**A**) Experimental protocol. F344 rats (5 weeks old, male) were treated with SUN (40 mg/kg BW, i.p.), LAP (40 mg/kg BW, i.p.), 5FU (30 mg/kg BW, i.p.), and CDDP (3 mg/kg BW, i.p.), with or without PTE (20 mg/kg BW, i.p.), twice a week for 4 weeks. (**B**) Heart weight, (**C**) SDS-MYL1, (**D**) mt4HNE, (**E**) mtGPX4, (**F**) GSH/GSSG ratio, (**G**) mtHO1, (**H**) mitochondrial total iron, (**I**) serum troponin T, and (**J**) time course of serum troponin T in 5FU-treated rats. (**K**) ATP. Error bar, standard deviation from 9 rats. Statistical difference was calculated by AVOVA. PTE: pterostilbene; SUN: sunitinib; LAP: lapatinib; 5FU: 5-fluorouracil; CDDP: cisplatin; C: control; BW: body weight; SDS-MYL1: sodium dodecyl sulfate-soluble myosin light chain-1; mt4HNE: mitochondrial 4-hydroxynonenal; mtGPX4: mitochondrial glutathione peroxidase-4; GSH: glutathione; GSSG: glutathione disulfide; mtHO1: mitochondrial haem oxygenase-1; BVD: biliverdin; OCR: oxygen consumption.

Finally, we investigated the effect of PTE on myocardial damage caused by anticancer drugs in a rat model. (Figure 7). F344 male rats were treated with four different anticancer drugs for 4 weeks with or without PTE (Figure 7A). Consequently, heart weight was reduced by all anticancer drugs (Figure 7B). In the myocardium, SDS-MYL1 levels decreased, mitochondrial lipid peroxide (mt4HNE) levels increased, mtGPX4 expression and the GSH/GSSG ratio decreased, mtHO1 expression increased, mitochondrial total iron levels increased, cardiac cell death marker (serum troponin T) levels increased, and ATP levels decreased (Figure 7C–J). This suggests that anticancer drugs induce ferroptosis and OXPHOS damage in the rat myocardium. In contrast, in rats treated with PTE, heart weight loss was prevented, and changes in SDS-MYL1, lipid peroxide, GPX4 protein, GSH/GSSG ratio, HO1 protein, mitochondrial total iron, serum troponin T, and ATP levels were rescued. These findings suggested that PTE inhibited anticancer-drug-induced myocardial injury in vivo.

## 3. Discussion

Our study suggests that anticancer drugs increase HO1 expression in the mitochondria of cardiomyocytes, resulting in mtFe deposition, ROS production, increases in lipid peroxide levels, decreases in GPX4 expression and redox potential, and consequent ferroptosis induction. Additionally, anticancer drugs impair mtDNA, resulting in an imbalance in the electron transport chain (ETC) complexes and decreased ATP production. In contrast, PTE inhibited the effects of anticancer drugs and exerted cardioprotective effects.

Our study showed that anticancer drugs induce ferroptosis in cardiomyocytes. Ferroptosis has been emphasized as a cause of myocardial damage in conditions other than those caused by anticancer drugs. Ferroptosis in the myocardium plays an important role in many pathological cardiac conditions, including heart failure, myocardial infarction/ischemia–reperfusion, and doxorubicin-induced cardiotoxicity [16,30,31,32,33,34]. In our study, anticancer drugs reduced GPX4 expression and the GSH/GSSG ratio, resulting in a redox imbalance. Together with its essential cofactor glutathione, GPX4 scavenges harmful byproducts of iron-dependent lipid peroxidation and protects cell membranes from damage [15]; thus, GPX4 deficiency increases susceptibility to ferroptosis [35]. Our data showed an increase in mitochondrial 4HNE levels, which is the major end product of polyunsaturated fatty acids oxidation and is commonly used as a marker of lipid peroxidation, which promotes ferroptosis by damaging cell membranes [36,37,38].

Our data showed that mtROS generation by anticancer drugs triggers a series of myocardial disorders. In cardiomyocytes, oxidative stress primarily induces ferroptosis and is less associated with apoptosis, necroptosis, and mitochondria-mediated necrosis [39]; this is consistent with the results of the inhibitor assay. Oxidative stress promotes glutathione depletion and GPX4 degradation in cardiomyocytes as well as increases lipid peroxidation [39]. Furthermore, iron overload increases the expression of HO1 and FCH as well as iron release by haem degradation [39,40], which are consistent with our results.

In our study, mtFe deposition was induced by all four anticancer drugs. DOX-induced myocardial disorder also showed mtFe deposition [16,41]. SUN, DOX, and LAP decrease the expression of mtNEET and ABCB8 with their platform in the mitochondria-associated endoplasmic reticulum membrane (MAM), PDZ Domain Containing 8 (PDZD8) [28,29]. However, their change was not found in H9c2 cells. The low expression of PDZD8 in the MAMs of normal cells may be one reason for this [29]. Therefore, the inhibitory effects of mtNEET and ABCB8, which have antiferroptotic effects on MAMs associated with PDZD8, are also likely to have a low contribution in cardiomyocytes [42,43,44,45].

Furthermore, our data indicated that anticancer drugs induce HO1 expression in the mitochondria of cardiomyocytes and promote haem degradation and deposition of its degradation product, iron (II), in mitochondria. The induction of HO1 expression induces ferroptosis in the myocardium, which is also observed in DOX-related cardiac disorders [16,46,47]. Thus, HO1 plays an important role in mtFe deposition and lipid peroxidation [16,48,49,50]. In contrast, the inhibition of HO1 activity by ZnPPIX inhibited anticancer-drug-induced ferroptosis. Similar findings have been reported in DOX-related myocardial injuries [16].

Herein, we discussed the role of ferroptosis in anticancer-drug-induced myocardial injury. However, in actual clinical practice, the prediction of CTRCD via elevated blood troponin T levels is limited in sensitivity [51], suggesting that ferroptosis might not be severe enough to cause heart failure. Our study suggests that impairment of myocardial energy metabolism caused by anticancer drugs may contribute to cardiac dysfunction. Mitochondrial dysfunction is widely recognized as a major factor in the progression of heart failure [41,52]. In our study, anticancer drugs caused an imbalance in ETC complex and decreased OXPHOS. Defects in one or more components of the ETC lead to reduced OXPHOS activity and decreased production of creatine phosphate and ATP in the myocardium [53]. Mitochondrial mtROS are the causes of these OXPHOS defects [53], whereas ETC disorders increase mtROS levels [54], which then cause mtDNA damage [55]. Thus, anticancer-drug-induced increases in mtROS levels cause mtDNA damage, which may form a malignant cycle leading to amplifying OXPHOS damage and further increasing mtROS production [56]. Thus, impairment of mitochondrial energy metabolism and ferroptosis are thought to be caused by the same conditions.

In our data, TFAM level was decreased by anticancer drugs. TFAM is an mtDNA-binding protein that is involved in repair; its levels decrease with DNA damage [57,58,59,60]. This suggests that mtDNA damage is induced by anticancer drugs targeting nucleic acids. DOX, which has been well studied, and nucleic-acid-disrupting anticancer drugs cause damage to mtDNA [60,61,62,63]. Interestingly, SUN and LAP, which do not directly damage DNA, have been suggested to induce mtDNA damage. Such molecularly targeted drugs may inhibit TFAM through inhibition of their on-target or off-target kinases [64,65]. This difference in direct or indirect impairment of mtDNA by signal inhibition may explain the difference in the reversibility of type 1 (nucleotide-disrupting drugs) and type 2 (molecularly targeted drugs) CTRCDs. In this study, we analyzed four anticancer drugs, two of which being molecularly targeted drugs and the other two being nucleotide-disrupting drugs. The former causes type 2 CTRCD and is often reversible, whereas the latter causes type 1 CTRCD and is often irreversible. In our study, their acute phase effect was elucidated as ferroptosis and impaired OXPHOS. To clarify this, longer observation using animal models is necessary. For example, in a rat model examining the cardiotoxicity of 5FU, an experimental period of 5 to 15 days has been reported [66,67]. We set an observation period of 4 weeks because in the 14-day study, although changes in ROS were observed, the induction of mitochondria iron, ferroptosis, and myocardial degeneration was insufficient. In this study, PTE ameliorated cancer-induced myocardial damage caused by anticancer drugs. PTE protects the myocardium from oxidative-stress-induced damage through activation of peroxisome proliferator-activated receptor coactivator-1α [23,27,68]. Furthermore, PTE suppresses inflammation by inhibiting HO1, while promoting wound healing by improving HO1 expression in diabetes [69,70]. This suggests that PTE may optimize intracellular ROS levels. Our study showed that even low-dose PTE, such as IC_20_, can suppress the cardiotoxicity caused by anticancer drugs. This expands the possibilities of PTE administration in clinical practice and helps overcome the low absorption of PTE from the gastrointestinal tract [71].

This study suggests that ferroptosis and OXPHOS inhibition associated with mtFe deposition are common cardiomyocyte injury pathways in CTRCD types 1 and 2. Furthermore, PTE may ameliorate both CTRCD types by inhibiting these pathways. In the future, extensive clinical investigations of PTE will be important.

## 4. Materials and Methods

### 4.1. Cell Culture

Embryonic rat-heart-derived H9c2 cardiomyoblasts were purchased from the American Type Culture Collection (Manassas, VA, USA) and cultured in DMEM supplemented with 10% fetal bovine serum (Sigma-Aldrich Chemical Co., St. Louis, MO, USA).

Cell growth was assessed using the 3-(4,5-dimethylthiazol-2-yl)-5-(3-carboxymethoxyphenyl)-2-(4-sulfophenyl)-2H-tetrazolium (MTS)-based Celltiter 96 aqueous one-solution cell proliferation assay kit (Promega Corporation, Madison, WI, USA), as previously described [61]. SUN, LAP, 5FU, CDDP (Wako Pure Chemical Corporation (Osaka, Japan)), ZnPPIX (Sigma-Aldrich), and PTE were purchased from MedChemExpress (Monmouth Junction, NJ, USA).

### 4.2. Mitochondrial Imaging

Mitochondrial function was examined using fluorescent probes. Cells were incubated with the probes for 30 min at 37 °C and then imaged using a BZ-X710 All-in-One fluorescence microscope (KEYENCE, Osaka, Japan). We used OxiORANGE (10 μM, Goryo Chemical, Sapporo, Japan) to assess mitochondrial hydroxyradicals, tetrathylrhodamine ethyl ester (TMRE, 200 nM, Sigma-Aldrich) to assess mitochondrial membrane potential, and mitoFerrogreen (20 nM, Dojindo, Kumamoto, Japan) to assess mtFe.

### 4.3. Protein Extraction

To prepare whole-cell lysates, cells were washed twice with cold PBS and harvested. The cells were then lysed with 0.1% NP-40-added RIPA buffer (Thermo Fisher Scientific, Tokyo, Japan) [72]. The mitochondrial fraction was extracted using the ReadiPrep mitochondrial/cytoplasmic fractionation kit (AAT Bioquest, Pleasanton, CA, USA) according to the manufacturer’s instructions. Protein assays were performed using the Protein Assay Rapid Kit (Wako).

### 4.4. Enzyme-Linked Immunosorbent Assay (ELISA)

ELISA kits were used to measure protein and iron concentrations (Table 1). The assay was performed using whole-cell lysates according to the manufacturer’s instructions.

### 4.5. Cell Death Inhibitor Assay

H9c2 cells were treated with SUN (1.2 μM), LAP (1.5 μM), 5FU (120 μM), or CDDP (10 μM) concurrent with the following cell death inhibitors for 24 h: ZVAD (10 μM) (Santa Cruz Biotechnology, Santa Cruz, CA, USA), NAC (1 mM) (Sigma), DFO (100 μM) (Cayman Chemicals, Ann Arbor, MI, USA), FRS (1 μM), and 4PBA (20 μM).

### 4.6. RNA Isolation

Total cellular RNA was isolated from each sample using the TRIzol reagent (Invitrogen, Waltham, MA, USA) and purified using the RNeasy Mini Kit (Qiagen, Hilden, Germany) according to the manufacturers’ protocol. The purified RNA was quantified using a NanoDrop ND-1000 spectrophotometer (Thermo Fisher Scientific).

### 4.7. Reverse Transcription–Polymerase Chain Reaction (RT-PCR)

RT-PCR was performed with 0.5 µg total RNA extracted from the three cell lines using the RNeasy kit (Qiagen, Germantown, MD, USA) to assess human and murine mRNA expression. The primer sets used are listed in Table 1 and were synthesized by Sigma Genosys (St. Louis, MO, USA). PCR products were electrophoresed on a 2% agarose gel and stained with ethidium bromide. ACTB mRNA was used as the internal control.

### 4.8. Quantification of BVD

BVD was quantified according to the method described by Suzuki [40,73]. The diazonium salt solution was prepared as follows: 10 mL of 5 mM 4-chloroanilin in 0.176 mM HCl was added to 72 mM of NaNO_2_. The mixture was allowed to stand at room temperature for 120 min until diazotization was complete. Mixture of 0.5 mL of the sample solution, 4.5 mL of methanol, and the diazonium salt solution was measured at an absorbance of 540 nm using a UV–visible spectrophotometer (UV-1280, Shimazu, Kyoto, Japan). A standard curve used for quantification was prepared by measuring the BVD standard sample (WAKO) in the same manner as described above.

### 4.9. Mitochondrial Stress Test (Seahorse Assay)

H9c2 cells were cultured in a growth medium in 6-well plates before the Seahorse assay with SUN (1.2 μM), LAP (1.5 μM), 5FU (120 μM), or CDDP (10 μM) for 24 h. OCR of 1 × 10^4^ viable H9c2 cells per well was measured using the Seahorse XFe24 Extracellular Flux Analyzer with Seahorse XF24 FluxPaks (Agilent Technologies, Chicopee, Canada). Seahorse assays were carried out as follows: OCR in pmol/min was measured before (basal OCR) and after successive injection of 1 µM oligomycin (ATP synthase inhibitor), 2 µM FCCP (carbonyl cyanide-p-trifluoromethoxy phenylhydrazone, an uncoupling protonophore), 1 µM rotenone (complex I inhibitor), and 5 µM antimycin A (complex III inhibitor). From the resulting data, we determined the OCR associated with respiratory ATP synthesis (oligomycin-sensitive), the maximum OCR in FCCP-uncoupled mitochondria, the rotenone-sensitive OCR attributable to uncoupled complex I activity, the antimycin-sensitive complex II/III activity, and the OCR by mitochondrial functions other than ATP synthesis, including OCR that is mitochondrial-membrane-potential-driven (proton leak), non-respiratory oxygen consumption, and the respiratory “spare capacity” (excess capacity of the respiratory ETC that is not being used in basal respiration).

### 4.10. Glycolytic Stress Test

The ECAR of H9c2 cells was measured using a modified glycolytic stress test on a Seahorse XFe24 Extracellular Flux Analyzer with Seahorse XF24 FluxPaks (Agilent Technologies, Chicopee, Canada). H9c2 cells were cultured in a growth medium in 6-well plates before the Seahorse assay with SUN (1.2 μM), LAP (1.5 μM), 5FU (120 μM), or CDDP (10 μM) for 24 h. H9c2 cells (1 × 10^4^ cells/well) were later plated in the XF base medium (Agilent Technologies, Chicopee, Canada) containing 200 mM L-glutamine and 5 mM HEPES, as recommended by the manufacturer for glycolytic assays. The sensor cartridge apparatus was rehydrated one day in advance by adding 1 mL XF Calibrant to each well and incubating at 37 °C until needed. The injection ports of the sensor cartridge apparatus were loaded with the following drugs, in chronological order of four injections, to meet the indicated final concentrations in the wells: 10 mM glucose, 1 µM oligomycin, 1 µM rotenone, and 5 µM antimycin A (combined injection) and 50 mM 2-deoxyglucose. Treatment with the rotenone/antimycin combination allowed the assessment of the impact of electron transport on ECAR by respiratory acidification coupled with the passage of some glycolytic pyruvate through the TCA cycle to supply respiration.

### 4.11. Animals

Five-week-old male F344 rats were purchased from SLC JAPAN (Shizuoka, Japan). The animals were maintained in a pathogen-free animal facility under a 12/12 h light/dark cycle in a temperature (22 °C)- and humidity-controlled environment. All procedures were performed in accordance with the institutional guidelines approved by the Committee for Animal Experimentation of Nara Medical University, Kashihara, Japan, following the current regulations and standards of the Japanese Ministry of Health, Labor, and Welfare (approval nos. 13082, 13126). Animals were acclimated to their housing for seven days before the start of the experiment. Rats were fed a CE-2 diet containing 5% crude fat, mainly derived from soybean oil (CLEA Japan, Inc., Tokyo, Japan).

Rats were treated with SUN (40/mg/kg body weight [BW], intraperitoneal [i.p.]) [29], LAP (40 mg/kg BW, i.p.) [74], 5FU (30 mg/kg BW, i.p.) [75], and CDDP (3 mg/kg BW, i.p.) [76] with or without PTE (20 mg/kg BW, i.p.) [27] twice a week for 4 weeks. The rats were euthanized by aortic blood removal under sevoflurane anesthesia (Maruishi Pharmaceutical Co. Ltd., Osaka, Japan). After euthanasia, hearts were excised, weighed, and used to analyze protein expression. Animal experiments were conducted in three studies using three rats per group.

### 4.12. Statistical Analysis

Statistical significance was calculated using ANOVA test using InStat software (version 3.1; GraphPad Software, Inc., La Jolla, CA, USA). The homogeneity of variance and normal distribution of each group was confirmed by Bartlett’s test. Data are expressed as the mean ± standard deviation of three independent experiments. Statistical significance was set at *p* < 0.05 (two-sided).

## Figures and Tables

**Table 1:** Primer sets and ELISA kits.

Primer Set					
Gene symbol	Gene bank ID	Forward primer (5′–3′)	Reverse primer (5′–3′)		
*HO1*	NM_002133.2	taagctggtgatggcttcct	atgatttcctgccagtgagg		
*FCH*	KR712044.1	gatgaattgtcccccaacac	gcttccgtcccacttgatta		
*mitoNEET*	BC007043.1	tccagaaagacaaccccaag	gcccacattgtctccagtct		
*ABCB8*	NM_001282291.2	cgtggggtctcgctttaact	cctgacactggcgagacaat		
*ACTB*	NM_001101.3	ggacttcgagcaagagatgg	agcactgtgttggcgtacag		
ELISA					
Protein	Catalog number	Range	Company		
4HNE	ab238538	1.2–200 μg/mL	Abcam, Cambridge, MA, USA		
GPX4	ARP-E4145	78.125–5000 pg/mL	Biocompare. South San Francisco, CA, USA		
Troponin T	CSB-E16443r	12.5–800 pg/mL	Cusabio Biotech Co., Ltd., Houston, TX, USA		
HO1	ab279414	15.63–1000 pg/mL	Abcam, Cambridge, MA, USA		
GSH/GSSG	ab138881	10 nM	Abcam, Cambridge, MA, USA		
Myogenin	LS-F16037	0.625–40 ng/mL	LS Bio, Shirley, MA, USA		
MYL1	MBS261960	0.312–20 ng/mL	MyBioSource, San Diego, CA, USA		
TFAM	OKCA01941	20–1800 pg/mL	Aviva System Biology, San Diego, CA, USA		
ETC Complex I	KLR2154	0.1–20 ng/mL	Krishgen Biosystems, Cerritos, CA, USA		
ETC Complex III	MBS2606461	0.1–20 ng/mL	Biocompare. South San Francisco, CA, USA		
ATP	LS-F24998	12.35–1000 ng/mL	LS Bio, Shirley, MA, USA		
Iron	MBS3801560	0.1 μmol/L	MyBioSource, San Diego, CA, USA		

*HO1*, haem oxygenase-1; *FCH*, ferrochelatase; *mitoNEET*, mitochondrial protein containing the Asn-Glu-Glu-Thr (NEET) sequence; *ABCB8*, ATP-binding cassette subfamily B member 8; *ACTB*, β-actin; 4HNE, 4-hydroxynonenal; GPX4, glutathione peroxidase-4; GSH/GSSG, glutathione/glutathione disulfide; MYL1, myosin light chain-1; TFAM, mitochondrial transcription factor A; ETC, electron transfer chain.

## Data Availability

Data are contained within the article.

## Abbreviation

CTRCD, cancer-therapy-related cardiac dysfunction; DOX, doxorubicin; GSH, glutathione; mtFe, mitochondrial iron (II); GPX4, glutathione peroxidase-4; PTE, pterostilbene; Nrf2, reduced nuclear factor erythroid 2; AMPK, AMP-activated protein kinase; SUN, sunitinib; LAP, lapatinib; 5FU, 5-fluorouracil; CDDP, cisplatin; 4HNE, 4-hydroxynonenal; ROS, reactive oxygen species; NAC, N-acetylcysteine; DFO, deferoxamine; FRS, ferrostatin 1; ZVAD, Z-VAD-FMK; 4PBA, 4-phenylbutyric acid; mtNEET, mitochondrial proteins containing the Asn-Glu-Glu-Thr (NEET) sequence; ABCB8, ATP-binding cassette subfamily B member 8; FCH, ferrochelatase; HO1, haem oxygenase-1; BVD, biliverdin; mtDNA, mitochondrial DNA; TFAM, mitochondrial transcription factor A; C-I, electron transfer chain complex I; C-III, electron transfer chain complex III; MMP, mitochondrial membrane potential; OCR, oxygen consumption ratio; ECAR, extracellular acidification rate; OXPHOS, oxidative phosphorylation; SDS-MYL1, sodium dodecyl sulfate-soluble myosin light chain-1; PUFA, polyunsaturated fatty acids; MAM, mitochondria-associated endoplasmic reticulum membrane; PDZD8, PDZ Domain Containing 8; ZnPPIX, zinc protoporphyrin IX; ETC, electron transport chain; Sirt-1, sirtuin-1.
